# Case of Rupture of the Carotid Artery, and Wounds of Several of Its Branches, Successfully Treated by Tying the Common Trunk of the Carotid Itself

**Published:** 1817-01

**Authors:** R. W. Coley, David Fleming

**Affiliations:** Member of the Royal College of Surgeons, a Surgeon in the Royal Navy, and a Resident Practitioner at Cheltenham; then Surgeon of His Majesty's Ship Tonnant


### Medico-Chirurgical
### JOURNAL AND REVIEW.

VOL. III.] JANUARY, 1817. [NO. 13.

### PART I.

### ORIGINAL COMMUNICATIONS.

—— quid utile ——

*To the Editors of the Medico-Chirurgical Journal & Review.*

GENTLEMEN,

Cheltenham, Nov. 14, 1816.

IN a book recently translated, entitled, " A Narrative of a Journey to London, in 1814; or, a Parallel of the English and French Surgery, by Philibert Joseph Roux," I was much pleased with the following handsome acknowledgment to English surgeons :—" It must be confessed, that the ligature of the common carotid and external iliac arteries, are incontestibly the two finest triumphs of modern surgery; we do not seem to have appreciated them sufficiently."—Without detracting, in the smallest degree, from the merits of the justly celebrated surgeons named by M. Roux, as having performed these operations, I am desirous of doing justice to the memory of a man, now no more, and lately belonging to a class of the profession whose merits are not duly appreciated, considering their opportunities for general and extensive practice.

By comparing dates, I have reason to believe, the honour of performing the first successful operation of tying the carotid artery, belongs to the late Mr. David

Fleming, a naval surgeon; and in proof of this assertion, I send you a concise statement of the case, drawn up from his Journal, by permission of Dr. Harness, of the Transport Board. I can bear testimony as to the result of the operation, for being First Assistant Surgeon of the Tonnant, I accompanied the man to Plymouth Hospital in March 1804. I am not aware why the case was not published by the late Mr. Fleming, as the success of the operation was much talked of by naval officers in general, belonging to the Channel Fleet.* The Tonnant was at the time one of the fleet, but on detached service.

I am, Gentlemen,

Your obedient Servant,

R. W. COLEY,

Member of the Royal College of Surgeons, a Surgeon in the Royal Navy, and a Resident Practitioner at Cheltenham.

---

*Case of Rupture of the Carotid Artery, and Wounds of several of its Branches, successfully treated by tying the common Trunk of the Carotid itself.* By the late DAVID FLEMING, Esq. then Surgeon of His Majesty's Ship Tonnant.

ON the 9th day of October, 1803, Mark Jackson, a servant on board His Majesty's Ship Tonnant, attempted to commit suicide. When called to him, I found that he had bled most profusely from a large wound across the throat. The bleeding vessels were secured by ligature as quickly as possible; and when the blood was cleared away, I was enabled to ascertain the extent of the injury, and the critical situation of my patient. No pulse could be felt at the wrists; the pupils were dilated; the temperature of the body considerably diminished; and, in fact, he had the appearance of a corpse from which life had recently departed. The wound was about four inches in length, dividing the trachea, between the thyroid and cricoid cartilages, the two superior thyroid arteries, the internal jugular vein, and grazed the outer and muscular coats of the carotid artery. The mouths of all these vessels (except the last) had been tied in the first instance, and I now

---

* We can account for the non-publication of the case. — Mr. Fleming sailed for the East Indies the year after the operation, and Dr. Johnson remembers to have heard him relate the case at Prince of Wales's Island, in 1805, and his intention to publish it on his return. He fell a victim to the climate shortly afterwards; and Dr. Johnson entirely forgot the circumstance, till the present paper brought it to his recollection.—ED.

brought the divided parts, as well as I could, into contact, and kept them so by securing the head well forward on the chest.

A small quantity of wine was got, with difficulty, into the stomach; and nearly two hours elapsed before he was sufficiently recovered to admit of being removed to the sick berth.

On the following morning he was low and faint. There was considerable tumefaction of the parts, which prevented his swallowing; and nourishing glysters were therefore thrown up. In the course of the day the pulse could be felt at the wrists, beating feebly about 100 in the minute. The wound put on a favourable appearance, and he enjoyed good nights. On the third day he was much troubled with a cough, which made me apprehensive of the rupture of the remaining coat of the artery; but all remained safe for the present, and the enemas were regularly administered.

On the 15th of October, the ligatures came away, and the favourable appearance of the wound induced me to hope that all would terminate well. But the cough increased in violence, occasioned by the discharge gaining admittance into the trachea; and on the evening of the 17th, during a violent paroxysm of coughing, the artery burst, and my poor patient was, in an instant, deluged with blood! The spectacle was rendered still more horrid, by the danger of suffocation, and the torrents of blood that issued from the mouth and nose! Several attempts were made to secure the artery, but all failed, from the degree of ulceration that extended along the tube, and syncope alone rescued the man, for a time, from actual death. In this dreadful situation, I concluded that there was but one step to take, with any prospect of success; namely, to cut down upon, and tie the carotid artery below the wound. I had never heard of such an operation being performed; but conceived that its effects might be less formidable, in this case, than in a person not previously reduced by hæmorrhage. My resolution taken, there was no time to lose; and I therefore made an incision along the anterior edge of the sternal portion of the cleido-mastoideus muscle; then separating the parts below with my fingers and the handle of a scalpel, till I brought the artery fairly into view, I passed a ligature round it, and put an éffectual stop to any farther loss of blood.

I had now time to reflect on what I had done. The neck appeared, from the cut inflicted by the patient himself, and my dissection, one continued wound, and presented an alarming aspect. For some time, my patient lay as if deprived of life. When, by the aid of stimulants, he was a little revived, the edges of the wound were brought together in the usual manner. I did not expect to find him alive the next morning. He was so, however; though he scarcely appeared to exist. His pulse could with difficulty be distinguished; and when spoken to, he appeared much confused.

The nutrient glysters were directed to be continued, and were all retained. His countenance gradually recovered animation; the heat of the body was slowly restored, and he passed quiet nights.

On the 20th of October, his pulse was 90, with increase of energy; and he seemed perfectly sensible. It is here worthy of remark, that till this morning he had no evacuation from the bowels during the space of eleven days.

On the 22d, the glysters were discontinued, as the power of deglutition was so far recovered that he could swallow wine and soup. The wound healed so rapidly, that on the 24th, the ligature was completely buried in sound granulations, and required to be removed by a cautious dissection. He had this day another evacuation, being the second since the operation.

The remainder of the case may be summed up in a few words. His strength remained for a long time impaired, from the great loss of blood; but being well supplied with nourishing diet, he was discharged from the sick list on the 20th of December, 1803, and retained as an attendant on the sick till March 1804, when he was sent to Plymouth Hospital to be invalided.

### Observations by the Editors.

This happened in 1803. In 1804, Mr. Abernethy's case was published, and therefore the claim of priority is at least divided between the two gentlemen. But the naval surgeon had great difficulties to contend with, and little assistance or advice. We believe that this interesting case presents the first and the only instance where the carotid artery was taken up after a rupture or wound of the artery itself, and life preserved. The transaction is recorded in the language of simplicity, and carries on its forehead the most convincing stamp of truth. Naval Surgery may justly be proud of this operation, which the hand of death buried for a time in oblivion. The departed spirit can no longer be gratified or grieved by honour or neglect; but justice is due to the dead as well as the living.

Palmam qui meruit ferat.

